# Comprehensive resistance evaluation of 15 blueberry cultivars under high soil pH stress based on growth phenotype and physiological traits

**DOI:** 10.3389/fpls.2022.1072621

**Published:** 2022-12-09

**Authors:** Hao Yang, Yaqiong Wu, Chunhong Zhang, Wenlong Wu, Lianfei Lyu, Weilin Li

**Affiliations:** ^1^ Co-Innovation Center for Sustainable Forestry in Southern China, Nanjing Forestry University, Nanjing, China; ^2^ Institute of Botany, Jiangsu Province and Chinese Academy of Sciences, Jiangsu Key Laboratory for the Research and Utilization of Plant Resources, The Jiangsu Provincial Platform for Conservation and Utilization of Agricultural Germplasm, Nanjing, China

**Keywords:** abiotic stress, phenotypic growth, osmotic regulators, antioxidant enzymes, photosynthesis

## Abstract

High soil pH is one of the main abiotic factors that negatively affects blueberry growth and cultivation. However, no comprehensive evaluation of the high soil pH tolerance of different blueberry cultivars has been conducted. Herein, 16 phenotypic and physiological indices of 15 blueberry cultivars were measured through pot experiments, and the high-pH soil tolerance coefficient (HSTC) was calculated based on these indices to comprehensively evaluate the high-soil-pH tolerance of plants. The results demonstrated that high soil pH stress inhibited blueberry 77.growth, and MDA, soluble sugar (SS), and soluble protein (SP) levels increased in leaves. Moreover, in all cultivars, CAT activity in the antioxidant system was enhanced, whereas SOD activity was reduced, and the relative expression levels of the antioxidant enzyme genes *SOD* and *CAT* showed similar changes. In addition, the leaf chlorophyll relative content (SPAD), net photosynthetic rate (P_n_), transpiration rate (E), and stomatal conductance (G_s_) decreased, while changes in the intercellular CO_2_ concentration (C_i_) were noted in different cultivars. Finally, according to the comprehensive evaluation value *D* obtained from the combination of principal component analysis (PCA) and membership function (MF), the 15 blueberry cultivars can be divided into 4 categories: high soil pH-tolerant type [‘Briteblue’ (highest *D* value 0.815)], intermediate tolerance type (‘Zhaixuan 9’, ‘Zhaixuan 7’, ‘Emerald’, ‘Primadonna’, ‘Powderblue’ and ‘Chandler’), low high soil pH-tolerant type (‘Brightwell’, ‘Gardenblue’, ‘Plolific’ and ‘Sharpblue’) and high soil pH-sensitive type [‘Legacy’, ‘Bluegold’, ‘Baldwin’ and ‘Anna’ (lowest *D* value 0.166)]. Stepwise linear regression analysis revealed that plant height, SS, E, leaf length, C_i_, SOD, and SPAD could be used to predict and evaluate the high soil pH tolerance of blueberry cultivars.

## Introduction

Blueberry, genus *Vaccinium* (family *Ericaceae*), is one of the most economically valuable fruit crops in the world and has been certified as one of five healthy fruits by the Food and Agriculture Organization (FAO) (Zang et al., 2022). Blueberry fruit has high levels of anthocyanins and other nutritional and healthcare-related functional substances that have strong antioxidant activity (Wu et al., 2022b). These substances can prevent cardiovascular disease (Cassidy et al., 2016), regulate immune ability (Kim et al., 2021), and intestinal microbial balance (Si et al., 2021), alleviate visual fatigue (Yao et al., 2015), and reduce blood glucose levels (Tian et al., 2021). These edible and health functions of blueberries have resulted in the continual expansion of their cultivation areas and scope globally (Wu et al., 2022a). By 2020, the cultivation area of blueberries in China has reached 66,400 ha (approximately 32.28% of the world’s cultivation area) (Yu et al., 2020). Nowadays, cultivated blueberries are mainly divided into three types according to plant growth size: highbush blueberry (*V. corymbosum*), rabbiteye blueberry (*V. virgatum*), and lowbush blueberry (*V. angustifolium*). Among them, highbush blueberry plant include southern highbush, northern highbush, and semi-highbush (hybrid type of highbush and lowbush blueberry) cultivars (Ghosh et al., 2018). Overall, southern highbush blueberries are the most widely planted in the world (Kumarihami et al., 2021).

Normally, blueberries are well known to grow best in acidic soil environments (optimal pH between 4.0 and 5.5) with high organic matter content and good drainage (Caspersen et al., 2016). Paradoxically, most field soil pH levels exceed the range suitable for blueberry growth (generally greater than 5.5) (Chen et al., 2019); thus, the high soil pH is a major abiotic stress factor for cultivated blueberry growth. In commercial production, high pH soil improvement is the basic requirement for the growth of cultivated blueberries; and high soil pH is currently reduced mainly by sulfur or acidic fertilizers, which are costly, poorly sustainable and environmentally unfriendly; overall, soil improvement is not an effective solution for field blueberry cultivation. Exploring the tolerance of different blueberry cultivars in high pH soils, as well as screening and cultivating blueberry cultivars that can grow in high pH soils, can effectively solve this problem.

Plant resistance is genetically related to the various morphological and physiological characteristics of the plant (Huseynova et al., 2016). Related studies have shown that high soil pH can cause plant chlorosis and inhibit phenotypic growth by reducing trace element absorption (El-Fouly et al., 2001; Turner et al., 2020). Meanwhile, high soil pH can cause oxidative stress in plants by increasing reactive oxygen species (ROS) and MDA contents while decreasing the activity of antioxidant enzymes (Kim et al., 2007; Valipour et al., 2020). Several studies have shown that high soil pH can damage blueberry plants’ photosynthetic, antioxidant, and osmotic adjustment systems, affect element absorption and transport, inhibit plant growth, delay flowering and flower bud differentiation, and reduce fruit yield and quality (Jiang et al., 2017; Jiang et al., 2019; Yang et al., 2022). Currently, many studies on the use of morphological and physiological indicators for plant resistance evaluation and cultivar screening have been reported. According to related studies, several blueberry cultivars have varying levels of tolerance for high soil pH (Darnell et al., 2015). However, a comprehensive evaluation of the tolerance of different blueberry cultivars to high soil pH has not been reported. Although some early studies involved evaluations of the high pH tolerance of blueberry plants (Chad et al., 1991) or *in vitro* screenings of blueberry plants with high pH tolerance levels (Finn et al., 1993; Tsuda et al., 2014; Yang et al., 2016; Darnell et al., 2020; Li et al., 2021), these studies were based on a single growth index.

The expression of stress resistance traits is a complex metabolic process controlled by multiple factors, and different plants have different levels of stress resistance. Therefore, it is difficult to accurately identify plant resistance based on a single trait parameter. However, when considering multiple indicators, there is a certain correlation between different indices, and the information will overlaps. Through a membership function (MF) combined with principal component analysis (PCA), the original related and complex indices can be transformed into simple, independent and comprehensive indices without losing the original information to evaluate the resistance of plants more objectively and comprehensively. This method has been widely used in plant resistance evaluation, superior plant identification, and screening (Sun et al., 2021; Weng et al., 2021; Zhao et al., 2022).

In the present study, a total of 15 blueberry cultivars, including southern highbush (5), northern highbush (4) and rabbiteye (6) cultivars, were selected for high soil pH stress tests. The tolerance of different blueberry cultivars to high soil pH levels was comprehensively evaluated based on 16 growth and physiological indices, and blueberry cultivars with strong resistance levels were screened. Furthermore, the physiological characteristics of different blueberry cultivars in response to high soil pH stress were investigated. This work aimed to provide a reference for blueberry cultivation and screening of resistant cultivars and lay a foundation for the rapid development of the blueberry industry.

## Materials and methods

### Plant materials

One-year-old cutting seedlings of 3 types of blueberry cultivars (n=15) suitable for planting and promotion in Jiangsu Province, China, were selected as the experimental materials, and detailed information is shown in **Table 1**. The plants were obtained from the Lishui Baima Blueberry Test Base in Nanjing, Jiangsu Province (31°36′5.66″N, 119°11′49.39″E), and the right to use the plants for these experiments was approved by Prof. Wu, a study collaborator.

**Table 1:** List of the 15 blueberry cultivars investigated.

No	Cultivar name	Parental relationship	Type
1	Baldwin	Ga.6-40 (Myers × Black Giant) × Tifblue	Rabbiteye blueberry (RB)
2	Briteblue	Ethel × Callaway	
3	Brightwell	(Ethel × Claraway) × Menditoo	
4	Gardenblue	Myers × Clara	
5	Powderblue	Tifblue × Menditoo	
6	Plolific	S2 × Centurion	
7	Anna	—	Southern highbush blueberry (SHB)
8	Primadonna	—	
9	Sharpblue	—	
10	Zhaixuan 7	—	
11	Zhaixuan 9	Florida 61-5 × Florida 62-4	
12	Bluegold	Blue Haven (Berkeley × 19-H) × ME-US5 (Ashworth × Bluecrop)	Northern highbush blueberry (NHB)
13	Chandler	Lateblue × Bluecrop	
14	Emerald	FL91-69 × NC1528	
15	Legacy	Elizabeth (Katharine × Jersey) × Scammell × US75 (Fla 4B × Bluecrop)	

### Plant cultivation and experimental design

**Figure 1:**
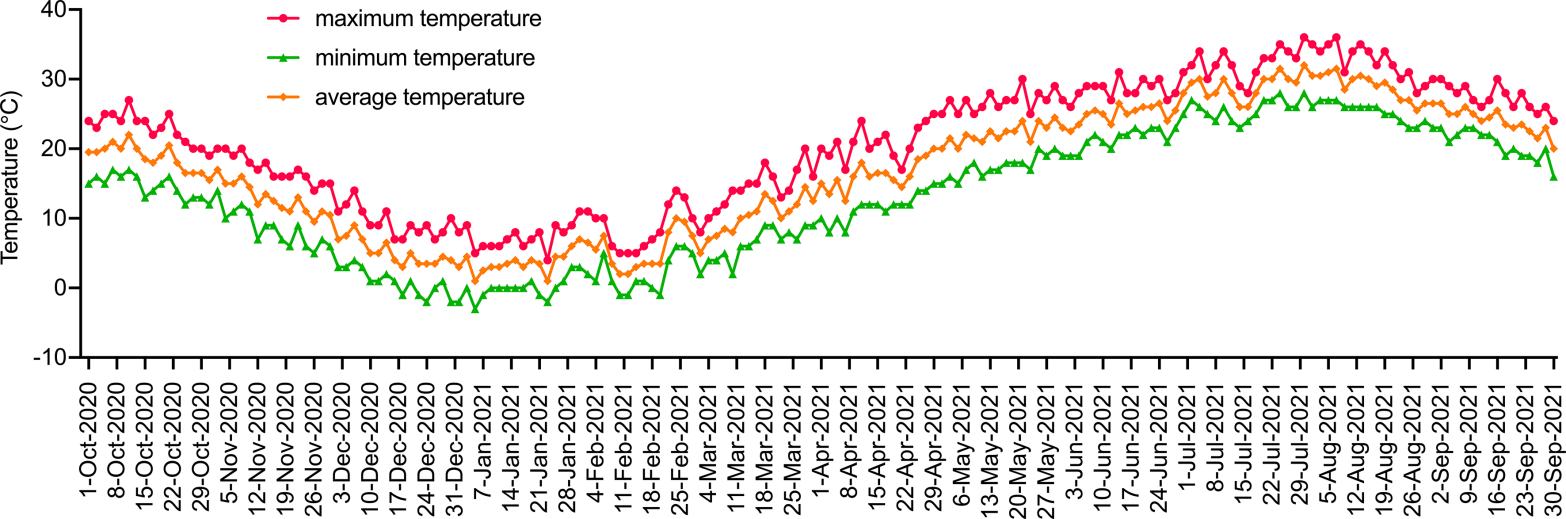
Climatic conditions of the test area from October 2020 to September 2021. Red, yellow and green represent the highest, lowest and average daily temperatures, respectively.

This experiment was conducted in a plastic high tunnel (with an automatic sprinkler system, without walls) at the nursery base of Jiangsu Institute of Botany, Chinese Academy of Sciences (32°3′16.01′′N, 118°49′51.44′′E) from November 2020 to September 2021. The experimental site is located in the north subtropical monsoon climate zone with an average annual temperature of 14.7°C, an average annual rainfall of 1000.4 mm, and an average frost-free period of 237 d. The climate changes in the test area during the test period are shown in **Figure 1**.

In November 2020, cutting seedlings of 15 blueberry cultivars with similar growth and no pests were transplanted into plastic pots (top diameter 17 cm, bottom diameter 12 cm, and height 14 cm), and the cultivation medium consisted of coarse peat, fine peat and perlite mixed in a 3:3:1 volume ratio with 0.5 kg medium used per pot. Two soil pH treatments were established in this experiment: the high soil pH stress treatment (pH 7.0) was prepared by mixing a specific amount of CaCO_3_ in the matrix, and the control treatment (pH 5.2) was prepared without the addition of CaCO_3_. Each treatment included 4 replicates, and each pot contained one blueberry cutting. All treatments were completely randomized. One month after transplantation, all the plants were uniformly pruned (only approximately 12 cm of the main stem remained). During the entire experimental period, tap water (pH ≈7.7) was used as the water source (maintaining the relative water content of the potting soil at 60% ± 5%). From November 2020 to March 2021, 400 mL of 1‰ blueberry-specific compound fertilizer solution was applied to each plant once a month. In addition, NaOH and C_6_H_8_O_7_·H_2_O aqueous solutions were used to maintain the pH at 5.2 (control) or at 7.0 (treatment), and the soil pH was regularly measured (pH meter AS-PH8, Aicevoos, China). The specific method was as follows: 400 mL of the corresponding pH solution was applied to each treatment every 3-5 days, and the soil pH changes were regularly measured to ensure that the pH error range remained within ± 0.1. At the end of August, the growth, and photosynthetic indices of the blueberry plants under different treatments were measured, and mature fresh leaves at the top of the plants were collected for physiological index analysis.

### Growth and leaf phenotypic indices

The plant height, crown width and main basal diameter of all blueberry cultivars in each treatment group were measured with a tape measure and digital Vernier calipers respectively. Five leaves with uniform growth in the middle of the primary lateral branches of blueberries were collected, with a total of 20 leaves collected from each treatment group, and the characteristics of the blueberry leaves (leaf length, width, and thickness) were measured with a digital Vernier caliper.

### Chlorophyll and photosynthetic characteristic indices

The relative chlorophyll content (SPAD value) in the top 3-5 mature leaves of the blueberry plants in each treatment group (a total of 10 leaves) was measured using a portable leaf chlorophyll meter (SPAD-502 plus, Minolta, Japan). In addition, three blueberry plants from each treatment group were randomly selected (a total of 8 leaves) for the determination of photosynthetic characteristics using a portable photosynthesis system (LI-6800, LI-COR Biosciences, U.S.) on September 6-9, 2021 (8:00-11:30 AM). For each plant, mature leaves with the top fully unfolded were selected to measure photosynthesis in a 2 cm^2^ fluorescence leaf chamber, and the parameters of the fluorescence leaf chamber were set as follows: the photosynthetically active radiation (PAR) was 1500 μmol·m^2^·s^-1^, the reference CO_2_ concentration was 400 μmol·mol^-1^, the leaf chamber temperature was 26°C, and the leaf chamber air humidity was 55%. In addition, the measurement indices included the net photosynthetic rate (P_n_), stomatal conductance (G_s_), intercellular CO_2_ concentration (C_i_), and transpiration rate (E).

### Physiological indices

Lipid peroxidation in stressed leaves was assessed by measuring malondialdehyde (MDA) through the thiobarbituric acid (TBA) method (Zeng et al., 2020). The Coomassie brilliant blue method (Bradford, 1976) and anthrone colorimetric method (Seifter & Dayton, 1950) were used to measure the contents of the osmotic regulator substances, including SP and SS, in the blueberry leaves under different treatments. For the extraction of leaf antioxidant enzymes, 0.1 g fresh leaves and 0.9 mL phosphate buffer (pH 7.4, 0.1 M) were mechanically homogenized in an ice-water bath and centrifuged at 10,000 rpm at 4°C for 10 min, and the supernatant was collected for testing. Superoxide dismutase (SOD) and catalase (CAT) activities in blueberry leaves were determined using the hydroxylamine and ammonium molybdate methods (Li et al., 2013). The amount of SOD corresponding to an SOD inhibition rate of 50% per g of tissue in 1 mL of the reaction solution was one SOD activity unit (U), and the amount of 1 μmol of H_2_O_2_ decomposed per mg of tissue protein per second was one CAT activity unit (U). Three biological replicates were performed for each measurement.

### High soil pH adaptability analysis

To eliminate the difference in measurement indices for the cultivars, high-pH soil tolerance coefficients (HSTCs) were used to comprehensively evaluate and analyze the tolerance of 15 blueberry cultivars to high soil pH stress (Szira et al., 2008). Then, the comprehensive index (CI) coefficient was obtained *via* factor analysis of the HSTCs, and each index was standardized to calculate the CI values for the different blueberry cultivars under high soil pH stress. The calculation formula was as follows:


(1) $$\text{HSTCs}=\frac{X_{pH\;7.0}}{X_{pH\;5.2}}$$

(2) $$\text{CI=}\sum_{i=1}^{\text{n}}\left[{\text{B}}_{\text{j}}{\;\times \;\mathrm{prin(m)}}_{\text{i}}\right]\text{, ( i = 1, 2, 3 ,……, n)}$$

where *X_pH 7.0_* and *X_pH 5.2_* are the values of the trait for different blueberry cultivars evaluated under soil pH 7.0 and pH 5.2 (CK), respectively. CI is the comprehensive index value, *B_j_* is the standardized value of the HSTCs for each index, and *prin(m)_i_* is the coefficient of the CI value.

Based on the PCA results and the CI value, the MF value and weight value of each CI were calculated based on Sun et al. (2021) and Niu et al. (2022). Finally, the comprehensive evaluation (*D*) value of the resistance of blueberry cultivars to high soil pH stress was calculated according to the method of Ding et al. (2018). The calculation formula was as follows:


(3) $${\text{U}}_{i}=\frac{C_{i}-C_{min}}{C_{max}-C_{min}}\;,\;\text{( i = 1, 2, 3 ,……, n)}$$

(4) $$W_{i}=\;\frac{P_{i}}{\sum_{i}^{n}P_{i}}\;,\;\text{( i = 1, 2, 3 ,……, n)}$$

(5) $$D=\sum_{i=1}^{\text{n}}\left[U_{i}\times W_{i}\right]\;,\;\text{( i = 1, 2, 3 ,……, n)}$$

*C_i_* is the *i*th CI value, and *C_max_* and *C_min_* are the maximum and minimum values of one given CI for all tested blueberry cultivars, respectively. *W_i_* is the importance of the *i*th CI in all CIs; *P_i_* is the contribution rate of the *i*th CI of each blueberry cultivar. The *D* value represents the comprehensive evaluation value of blueberry resistance under high soil pH stress.

### Gene expression analysis

The total RNA of frozen fresh blueberry leaves was extracted using a BioTeke Plant Total RNA Extraction Kit (RP3301, Beijing, China) and reversed into cDNA using PrimeScript RT Master Mix (Perfect Real Time) (TaKaRa, Japan). Then, quantitative real-time PCR was carried out with an Applied Biosystems 7500/7500 Fast Real-Time PCR System (Thermo Fisher Scientific) to evaluate the expression of *VcSOD1*, *VcSOD2*, *VcSOD3*, *VcCAT1*, *VcCAT2* and *VcCAT3* using TB Green Premix Ex Taq II (Tli RNaseH Plus) (TaKaRa, Japan) in a 15 µL reaction system. Primer sequence information is shown in **Supplementary Table 1**. The qRT−PCR reaction procedure was 95°C for 30 s, followed by 40 cycles at 95°C for 5 s and 60°C for 34 s. GAPDH was selected as the reference gene and the relative gene expression was calculated using the 2^−ΔΔCt^ method (Livak & Schmittgen, 2001). Three biological replicates were used in this experiment.

### Statistical analysis

One-way analysis of variance (ANOVA) of all the test data was performed SPSS 25.0 software (IBM Corp., Armonk, NY, USA), and Duncan’s multiple comparisons test was used to test the significance of differences. For each cultivar, the mean HSTCs were used for a subsequent PCA and MF calculation. The *D* value of blueberry resistance was analyzed using a systematic clustering method. Pearson correlation analysis and linear regression analysis were used to determine the relationship between the *D* value and the HSTCs. All statistical data were plotted and constructed using GraphPad Prism 8 software (GraphPad Software, San Diego, CA, USA) and Origin 2022 (Origin Lab Inc., USA).

## Results

### Plant growth index of blueberry

**Figure 2:**
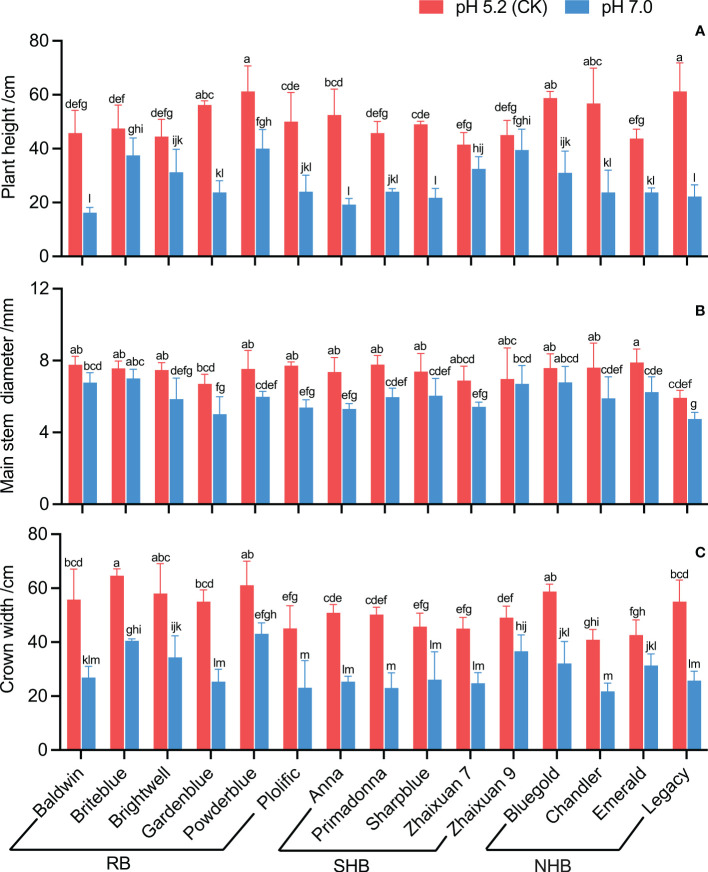
Effects of high soil pH stress on the growth characteristics of 15 blueberry seedling cultivars. The plant height **(A)**, main stem diameter **(B)**, and crown width **(C)**. Columns with different letters significantly (p<0.05) differed based on Duncan’s multiple range test.

The results showed that high soil pH stress inhibited the growth of blueberry cultivars to varying degrees (**Figure 2**). In a high soil pH of 7.0, the plant height of cultivars decreased significantly except that of ‘Zhaixuan 9’, and the plant height of ‘Baldwin’, ‘Legacy’ and ‘Anna’ decreased by 64.48%, 63.67% and 63.33%, respectively (**Figure 2A**). The main basal diameter growth of ‘Baldwin’, ‘Briteblue’, ‘Zhaixuan 9’ and ‘Bluegold’ was inhibited under the soil pH stress treatment, but no significant difference from the CK treatment was observed. Of the cultivars, ‘Legacy’ had the smallest main basal diameter (4.75 mm), and ‘Anna’ and ‘Plolific’ had large decline rates of 28.03% and 30.23%, respectively (**Figure 2B**). The crown growth of blueberry cultivars significantly decreased under high soil pH stress treatment; ‘Powderblue’ had the largest crown (43.13 cm) followed by ‘Briteblue’ and ‘Zhaixuan 9’. Moreover, the crown growth of ‘Primadonna’, ‘Gardenblue’, ‘Legacy’, ‘Baldwin’ and ‘Anna’ all decreased by greater than 50% (range, 50.12% to 54.23%) (**Figure 2C**).

### Blueberry leaf morphological features

**Figure 3:**
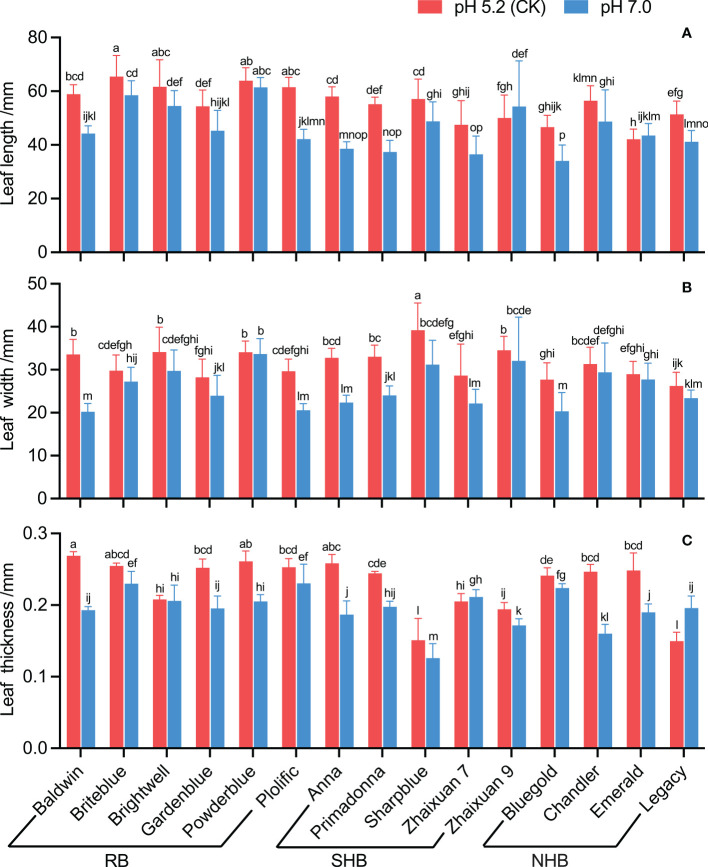
Effects of high soil pH stress on the leaf traits of 15 blueberry seedling cultivars. The leaf length **(A)**, leaf width **(B)**, and leaf thickness **(C)**. Columns with different letters significantly (p < 0.05) differed based on Duncan’s multiple range test.

Based on the leaf morphology feature analysis, the leaf length decreased in all cultivars under high soil pH stress except ‘Emerald’ and ‘Zhaixuan 9’. ‘Bluegold’ had the shortest length (34.05 mm), and the leaf growth decline rate of ‘Anna’ was the greatest (33.54%) (**Figure 3A**). In addition, the leaf width of blueberry cultivars showed different degrees of reduction under a high soil pH of 7.0. ‘Baldwin’ had the smallest leaf width (20.23 mm), which decreased by 39.72% (**Figure 3B**). Regarding leaf thickness, under high soil pH stress, the leaf thickness of ‘Legacy’ and ‘Zhaixuan 7’ increased, but that of the other cultivars decreased. In addition, the leaf thickness of ‘Chandler’ and ‘Anna’ decreased significantly by 35.07% and 27.80%, respectively (**Figure 3C**).

### Blueberry leaf physiological characteristics

**Figure 4:**
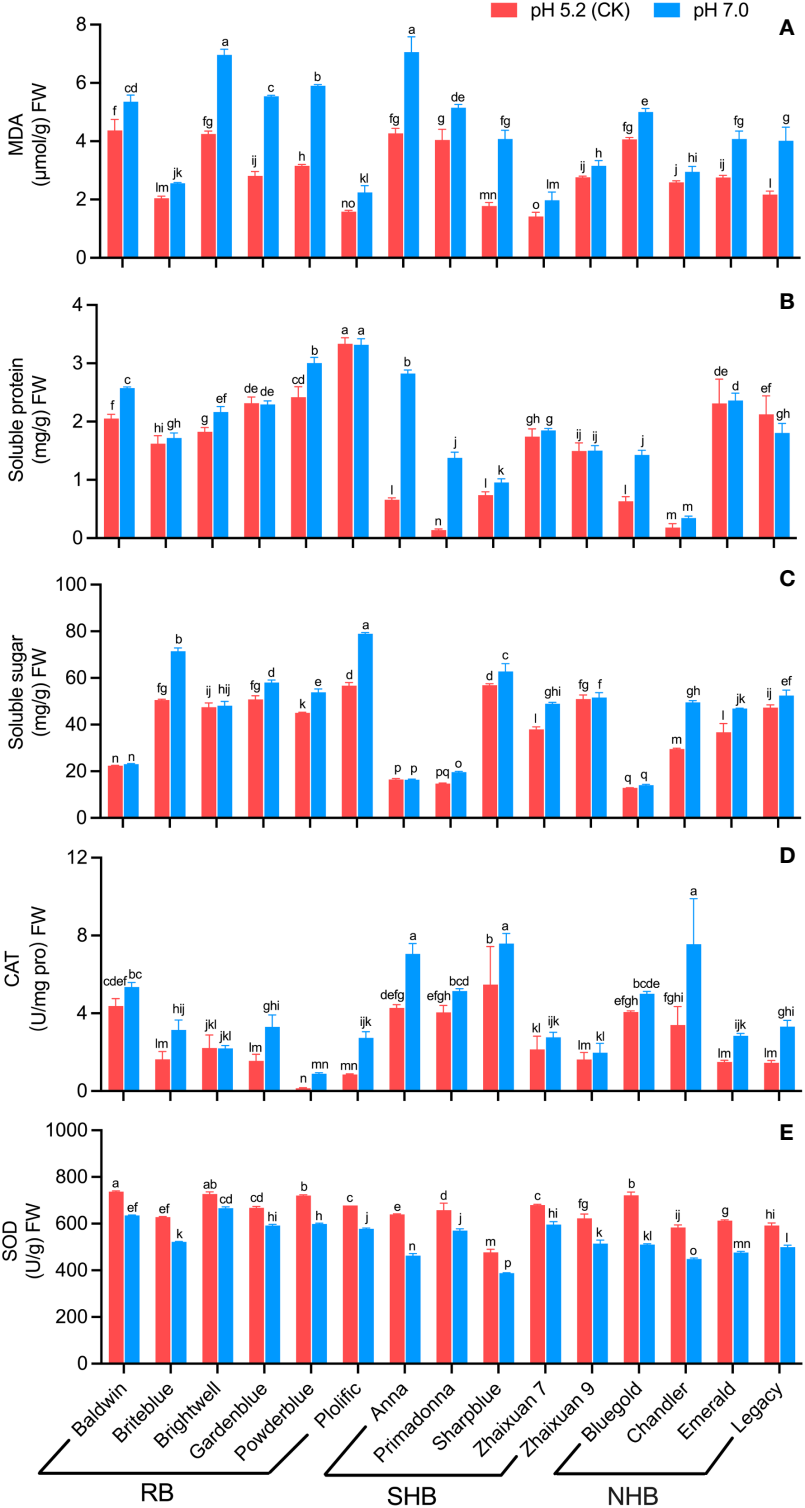
Effects of high soil pH stress on the leaf physiological characteristics of 15 blueberry seedling cultivars. The content of MDA **(A)**, soluble protein **(B)**, and soluble sugar **(C)**, the enzyme activity of CAT **(D)** and SOD **(E)**. Columns with different letters significantly (p < 0.05) differed based on Duncan’s multiple range test.

High soil pH stress resulted in a significant increase in MDA content in blueberry leaves compared to leaves under CK conditions (**Figure 4A**). Among the cultivars, ‘Anna’ had the highest MDA content (7.05 μmol/g FW), and ‘Zhaixuan7’ had the lowest MDA content (1.97 μmol/g FW). The MDA content in ‘Sharpblue’ was 1.30 times that noted in leaves in the CK group; the MDA content in ‘Gardenblue’, ‘Powderblue’ and ‘Legacy’ increased by 97.25%, 87.15% and 84.78%, respectively, whereas values in ‘Chandler’ and ‘Zhaixuan 9’ increased gradually (14.05% and 14.25%, respectively). The above analysis analyses revealed that different blueberry cultivars experienced significantly different degrees of membrane lipid damage under high soil pH stress, resulting in different degrees of damage to their leaves.

Under high soil pH stress treatment, the SP content in leaves of ‘Gardenblue’, ‘Plolific’ and ‘Legacy’ decreased compared with levels in CK group leaves, and the other cultivars showed different degrees of increase (**Figure 4B**). ‘Plolific’ had the highest SP content (3.32 mg/g FW), and ‘Chandler’ had the lowest SP content (0.35 mg/g FW). The SS contents in ‘Primadonna’, ‘Anna’ and ‘Bluegold’ leaves were 8.86, 3.28 and 1.27 times higher than that in CK leaves, respectively. The SS content in ‘Chandler’ and ‘Briteblue’ increased significantly (67.77% and 41.30%, respectively). No significant differences were noted among ‘Baldwin’, ‘Brightwell’, ‘Anna’ and ‘Bluegold’ between high soil pH 7.0 and CK conditions (**Figure 4C**). The above analyses showed that osmoregulatory substances were related to the ability of different blueberry cultivars to adapt to high soil pH stress.

In comparison to CK conditions, in the high soil pH stress treatment, the CAT activity of blueberry leaves of different cultivars increased (**Figure 4D**), whereas SOD activity was inhibited (**Figure 4E**). ‘Briteblue’ showed the highest CAT activity (7.58 U/mg pro FW), and ‘Powderblue’ had the lowest CAT activity at 0.89 U/mg pro FW. The SOD activity in ‘Anna’ and ‘Bluegold’ leaves decreased significantly by 27.64% and 29.26%, respectively, and the ‘Brightwell’ decrease rate was the lowest at 8.35%, whereas moderate values were noted for the other cultivars. The above analyses showed that these two antioxidant enzymes played a specific role in blueberry adaptation to a high soil pH environment.

### Blueberry leaf photosynthetic and physiological characteristics

Overall, high soil pH stress inhibited the photosynthesis in blueberry plants (**Table 2**). The SPAD value for blueberry plants decreased under high soil pH stress compared with that for plants in the CK group, and the decreased rates for ‘Bluegold’, ‘Anna’, ‘Baldwin’ and ‘Legacy’ were more than 35%. ‘Plolific’ had the lowest reduction rate at 17.23%. Under high soil pH stress, the P_n_ of blueberry plants was inhibited to different degrees. ‘Chandler’ (66.66%) and ‘Brightwell’ (65.02%) had greater reduction rates, whereas that of ‘Emerald’ was the lowest (26.63%). The E and G_s_ values increased in ‘Primadonna’ compared with those parameters in CK plants, and in other cultivars, those values were lower than in CK plants. Finally, the E values for ‘Baldwin’, ‘Brightwell’ and ‘Legacy’ were significantly lower than that for CK (p<0.05), and the rate of decrease exceeded 70%. However, ‘Zhaixuan 7’ and ‘Briteblue’ showed smaller decreases in E (8.79% and 11.68%, respectively). The C_i_ in ‘Legacy’ increased by 20.76% compared with that in CK plants, while the C_i_ in ‘Briteblue’ decreased by 27.75%. The above analyses showed that different blueberry cultivars experience different degrees of damage to their photosynthetic systems under high soil pH stress.

**Table 2:** Effects of high soil pH stress on the leaf photosynthetic physiological characteristics of 15 blueberry seedling cultivars.

Cultivar	Type	SPAD		P_n_ (µmol·m-²·s-¹)		E (mmol·m-²·s-¹)		C_i_ (µmol·mol-¹)		G_s_ (mol·m-²·s-¹)	
		pH 5.2 (CK)	pH 7.0	pH 5.2 (CK)	pH 7.0	pH 5.2 (CK)	pH 7.0	pH 5.2(CK)	pH 7.0	pH 5.2 (CK)	pH 7.0
Baldwin	Rabbiteye blueberry (RB)	48.40 ± 4.02d	32.35 ± 3.76cde	10.17 ± 1.26b	4.28 ± 1.17a-d	4.35 ± 0.41b	0.66 ± 0.41h	302.65 ± 13.43a	322.20 ± 34.39a	0.20 ± 0.02b	0.03 ± 0.02e
Briteblue		49.90 ± 2.84cd	31.14 ± 4.19cde	7.84 ± 1.62d-g	4.42 ± 1.75a-d	1.68 ± 0.61g	1.48 ± 0.69ef	209.67 ± 35.11c	290.22 ± 22.66bc	0.07 ± 0.03g	0.06 ± 0.03cd
Brightwell		52.85 ± 4.25c	35.53 ± 8.00bc	9.50 ± 0.78bcd	3.32 ± 0.70de	3.71 ± 0.37bc	0.98 ± 0.29fgh	305.49 ± 7.18a	263.74 ± 30.40cd	0.19 ± 0.02bc	0.05 ± 0.01de
Gardenblue		40.27 ± 4.34f	35.48 ± 6.26bc	7.74 ± 1.11efg	3.63 ± 0.75cde	2.65 ± 0.54def	1.39 ± 0.47efg	283.66 ± 19.89ab	255.57 ± 34.80d	0.13 ± 0.03d-g	0.06 ± 0.02d
Powderblue		47.82 ± 4.28d	38.35 ± 5.70b	9.01 ± 1.62b-f	5.09 ± 1.36ab	2.19 ± 0.62efg	1.08 ± 0.37e-h	238.10 ± 28.81c	220.76 ± 41.21e	0.10 ± 0.03fg	0.05 ± 0.02de
Plolific		53.69 ± 3.55b	35.87 ± 4.64bc	8.07 ± 1.1c-f	3.49 ± 0.28cde	2.79 ± 1.31c-f	0.89 ± 0.16gh	285.23 ± 36.98ab	248.81 ± 20.76d	0.15 ± 0.08c-f	0.04 ± 0.01de
Anna	Southern highbush blueberry (SHB)	47.52 ± 2.61d	29.17 ± 3.85de	7.73 ± 2.06efg	4.24 ± 0.81a-d	2.47 ± 1.21d-g	1.23 ± 0.39efg	284.68 ± 28.60ab	260.54 ± 32.60d	0.12 ± 0.06efg	0.06 ± 0.02d
Primadonna		44.30 ± 2.63e	32.78 ± 7.74cde	8.20 ± 2.97c-f	4.83 ± 1.56abc	2.31 ± 0.81efg	2.58 ± 0.56ab	271.66 ± 29.74b	321.61 ± 23.16a	0.11 ± 0.04efg	0.12 ± 0.03a
Sharpblue		44.55 ± 2.13e	35.71 ± 4.86bc	6.26 ± 0.23gh	3.90 ± 0.52bcd	2.12 ± 0.80efg	0.95 ± 0.46gh	290.57 ± 26.80ab	213.01 ± 18.02e	0.10 ± 0.04fg	0.04 ± 0.02de
Zhaixuan 7		50.35 ± 4.02d	39.27 ± 7.38ab	9.59 ± 3.33bc	5.55 ± 2.33a	3.27 ± 0.81cd	2.99 ± 1.03a	283.96 ± 29.57ab	329.25 ± 16.59a	0.16 ± 0.04b-e	0.14 ± 0.05a
Zhaixuan 9		48.96 ± 3.55d	36.21 ± 3.08bc	6.20 ± 0.98gh	2.32 ± 1.61e	3.00 ± 0.90cde	1.54 ± 0.62de	300.34 ± 30.20ab	325.54 ± 26.38a	0.13 ± 0.04def	0.06 ± 0.03cd
Bluegold	Northern highbush blueberry (NHB)	57.25 ± 2.75a	34.26 ± 7.90bcd	9.06 ± 1.87b-e	3.83 ± 1.60bcd	3.59 ± 1.59bc	1.10 ± 0.59e-h	281.95 ± 44.84ab	257.58 ± 25.85d	0.18 ± 0.09bcd	0.05 ± 0.03de
Chandler		49.39 ± 2.48d	28.59 ± 3.15e	13.00 ± 1.83a	4.36 ± 1.23a-d	5.73 ± 1.01a	2.04 ± 0.49cd	311.23 ± 20.19a	305.82 ± 22.25ab	0.30 ± 0.07a	0.09 ± 0.02bc
Emerald		49.06 ± 4.51d	44.44 ± 5.27a	7.30 ± 1.86fg	5.36 ± 0.81a	2.53 ± 1.04d-g	2.13 ± 0.63bc	286.41 ± 24.55ab	290.02 ± 23.32bc	0.12 ± 0.05efg	0.10 ± 0.03b
Legacy		43.06 ± 2.38ef	27.68 ± 7.73e	5.21 ± 0.39h	3.10 ± 0.62de	1.94 ± 1.09fg	0.58 ± 0.37h	286.82 ± 35.95ab	238.53 ± 41.94de	0.10 ± 0.06fg	0.03 ± 0.02e

SPAD, chlorophyll relative content; P_n_, net photosynthetic rate; E, transpiration rate; C_i_, intercellular CO_2_ concentration; G_s_, stomatal conductance. The data are reported as the means ± SDs. Different letters for values in the same column/trait indicate significant differences for each cultivar in the same treatment (p<0.05).

### Correlation analysis

**Figure 5:**
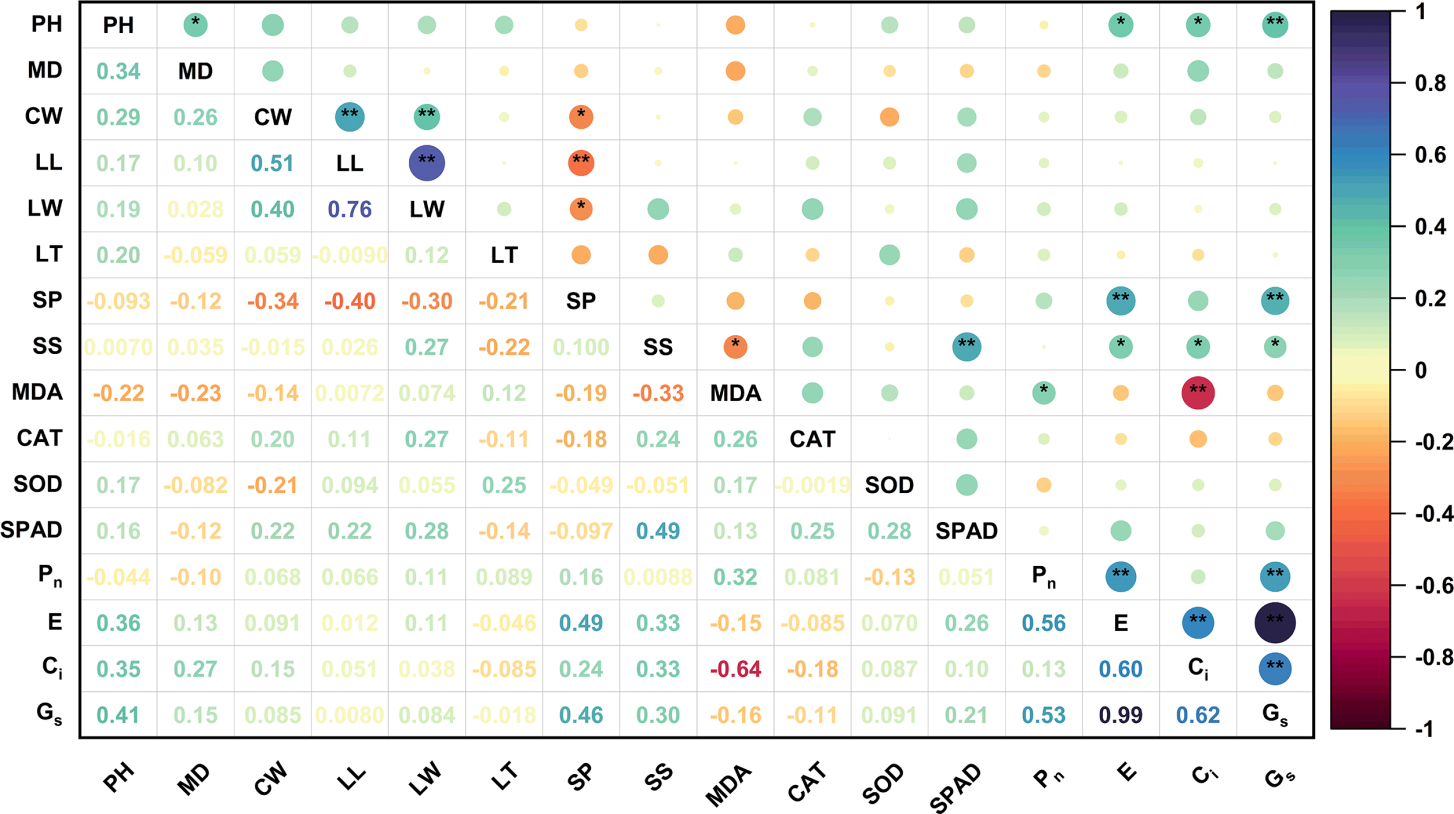
Correlation matrix of the HSTCs of the growth and leaf physiological indices of 15 blueberry seedling cultivars under high soil pH stress. The results were derived from the Pearson correlation analysis. PH, plant height; MD, main stem diameter; CW, crown width; LL, leaf length; LW, leaf width; LT, leaf thickness; SP, soluble protein; SS, soluble sugar; MDA, malondialdehyde; SOD, superoxide dismutase; CAT, catalase; SPAD, chlorophyll relative content; P_n_, net photosynthetic rate; E, transpiration rate; C_i_, intercellular CO_2_ concentration; G_s_, stomatal conductance. The circle size and color intensity are proportional to the value of each correlation coefficient. Positive and negative correlations are displayed in purple and red, respectively. * represents a significant correlation at the 0.05 level, and ** represents a significant correlation at the 0.01 level.

To eliminate the differences among the different indices and better understand the relationship between the different effects of high soil pH stress on blueberry cultivars, HSTCs were used for data analysis. Significant differences were found in the responses of different blueberry cultivars to high soil pH stress, except for the MD value (**Table S2**). Under high soil pH stress, blueberry PH was positively correlated with the MD, E, C_i_, and G_s_ (**Figure 5**). SP was positively correlated with E and G_s_ but negatively correlated with LW, LL, and CW. SS was significantly positively correlated with SPAD, E, C_i_, and G_s_ and negatively correlated with MDA. C_i_ was also negatively correlated with MDA. The above analyses shows that blueberry growth is closely related to osmotic adjustment substances, the antioxidant system and photosynthesis. The synergistic effect of these systems can improve the adaptability of blueberries in high soil pH environments.

### PCA of 16 physiological growth indices

PCA was used to extract the relative values of 16 physiological growth indices. Based on eigenvalues greater than 1, 5 principal components (PCs) were extracted. The sum of their eigenvalues was 13.14, and the cumulative variance contribution rate was 82.12% (**Table 3**). PC1 (25.59%) mainly reflected the effect of high soil pH stress on the growth indices of PH, CW, LL, LW (positive loading) and SP (negative loading). PC2 (19.66%) was significantly correlated with the photosynthetic indices P_n_, E and G_s_ with positive loading. PC3 (16.12%) included MD and C_i_ with significantly positive loading and MDA with negative loading. PC4 (13.85%) included SS, CAT, and SPAD with positive loading. PC5 (9.89%) included LT and SOD with positive loading.

**Table 3:** PCA based on the HSTCs of the growth and leaf physiological indices of 15 blueberry seedling cultivars under high soil pH stress.

Trait	Component				
	1	2	3	4	5
Plant height	**0.643**	0.260	0.478	0.017	0.371
Main stem diameter	0.561	-0.083	**0.596**	-0.351	-0.046
Crown width	**0.923**	0.122	0.069	-0.016	-0.137
Leaf length	**0.947**	-0.033	0.025	0.089	0.023
Leaf width	**0.810**	0.059	-0.122	0.336	0.063
Leaf thickness	0.070	0.026	-0.174	-0.437	**0.624**
Soluble protein (SP)	**-0.563**	0.523	0.186	0.044	-0.175
Soluble sugar (SS)	-0.039	0.188	0.212	**0.825**	-0.125
Malondialdehyde (MDA)	0.032	-0.025	**-0.905**	-0.077	0.192
Catalase (CAT)	0.292	-0.106	-0.443	**0.517**	-0.131
Superoxide dismutase (SOD)	-0.025	-0.003	0.015	0.204	**0.888**
Chlorophyll relative content (SPAD)	0.216	0.134	-0.076	**0.832**	0.296
Net photosynthetic rate (P_n_)	0.112	**0.767**	-0.491	-0.129	-0.178
Transpiration rate (E)	0.033	**0.960**	0.165	0.181	0.075
Intercellular CO_2_ concentration (C_i_)	0.093	0.540	**0.735**	0.154	0.055
Stomatal conductance (G_s_)	0.049	**0.955**	0.189	0.135	0.115
Eigenvalues	3.615	3.146	2.580	2.216	1.583
% of Variance	22.593	19.660	16.123	13.853	9.891
Cumulative %	22.593	42.253	58.375	72.228	82.120

Bold numbers indicate eigenvalues are significant ≥|0.5|.

### Comprehensive evaluation of high soil pH stress tolerance

To comprehensively evaluate the tolerance of 15 blueberry cultivars to high soil pH levels, the CI values of 5 PCs were calculated *via* PCA, and the CI values were transformed using the MF calculation formula. The *D* value of blueberry resistance under high soil pH stress was obtained by a weight calculation (**Table 4**). The greater the *D* value, the stronger the tolerance. The results showed that the 15 blueberry cultivars were ranked as follows: ‘Briteblue’ > ‘Zhaixuan 9’ > ‘Zhaixuan 7’ > ‘Emerald’ > ‘Primadonna’ > ‘Powderblue’ > ‘Chandler’ > ‘Brightwell’ > ‘Gardenblue’ > ‘Plolific’ > ‘Sharpblue’ > ‘Legacy’ > ‘Bluegold’ > ‘Baldwin’ > ‘Anna’.

**Table 4:** Comprehensive index value (*C_i_*), membership function value (*U_i_*), weight (*W_i_*), comprehensive evaluation value (*D* value) and ranking of resistance ability for each comprehensive index value for 15 blueberry seedling cultivars under high soil pH stress.

Cultivar	*C_1_*	*C_2_*	*C_3_*	*C_4_*	*C_5_*	*U_1_*	*U_2_*	*U_3_*	*U_4_*	*U_5_*	*D* value	Rank
Baldwin	-3.620	-3.882	1.352	-2.829	-0.966	0.127	0.000	0.651	0.123	0.362	0.227	14
Briteblue	4.094	4.521	4.221	2.177	0.975	0.777	0.778	1.000	0.784	0.722	0.815	1
Brightwell	1.471	-3.352	-0.360	-1.401	2.475	0.556	0.049	0.442	0.312	1.000	0.424	8
Gardenblue	-1.442	-1.261	-2.570	1.274	1.130	0.310	0.243	0.173	0.665	0.750	0.380	9
Powderblue	4.817	-0.425	-2.716	2.513	-0.303	0.838	0.320	0.155	0.829	0.485	0.536	6
Plolific	-3.243	-2.372	-1.690	2.263	0.566	0.158	0.140	0.280	0.796	0.646	0.344	10
Anna	-5.123	-0.164	-1.660	-2.483	-2.915	0.000	0.344	0.284	0.169	0.000	0.166	15
Primadonna	-5.120	6.917	2.538	1.588	0.155	0.000	1.000	0.795	0.707	0.570	0.583	5
Sharpblue	-0.223	-0.984	-3.990	-0.763	-0.013	0.413	0.268	0.000	0.396	0.538	0.309	11
Zhaixuan 7	0.132	3.997	2.042	0.331	2.194	0.443	0.730	0.735	0.540	0.948	0.645	3
Zhaixuan 9	6.739	-0.294	4.172	-1.079	0.958	1.000	0.332	0.994	0.354	0.719	0.695	2
Bluegold	-1.962	-2.493	1.349	-3.760	-2.250	0.266	0.129	0.650	0.000	0.123	0.246	13
Chandler	0.513	-1.947	1.486	3.809	-1.978	0.475	0.179	0.667	1.000	0.174	0.494	7
Emerald	4.170	3.905	-0.601	1.081	-1.374	0.783	0.721	0.413	0.640	0.286	0.611	4
Legacy	-1.203	-2.166	-3.572	-2.721	1.345	0.330	0.159	0.051	0.137	0.790	0.257	12
*W_i_*						0.275	0.239	0.196	0.169	0.120		

**Figure 6:**
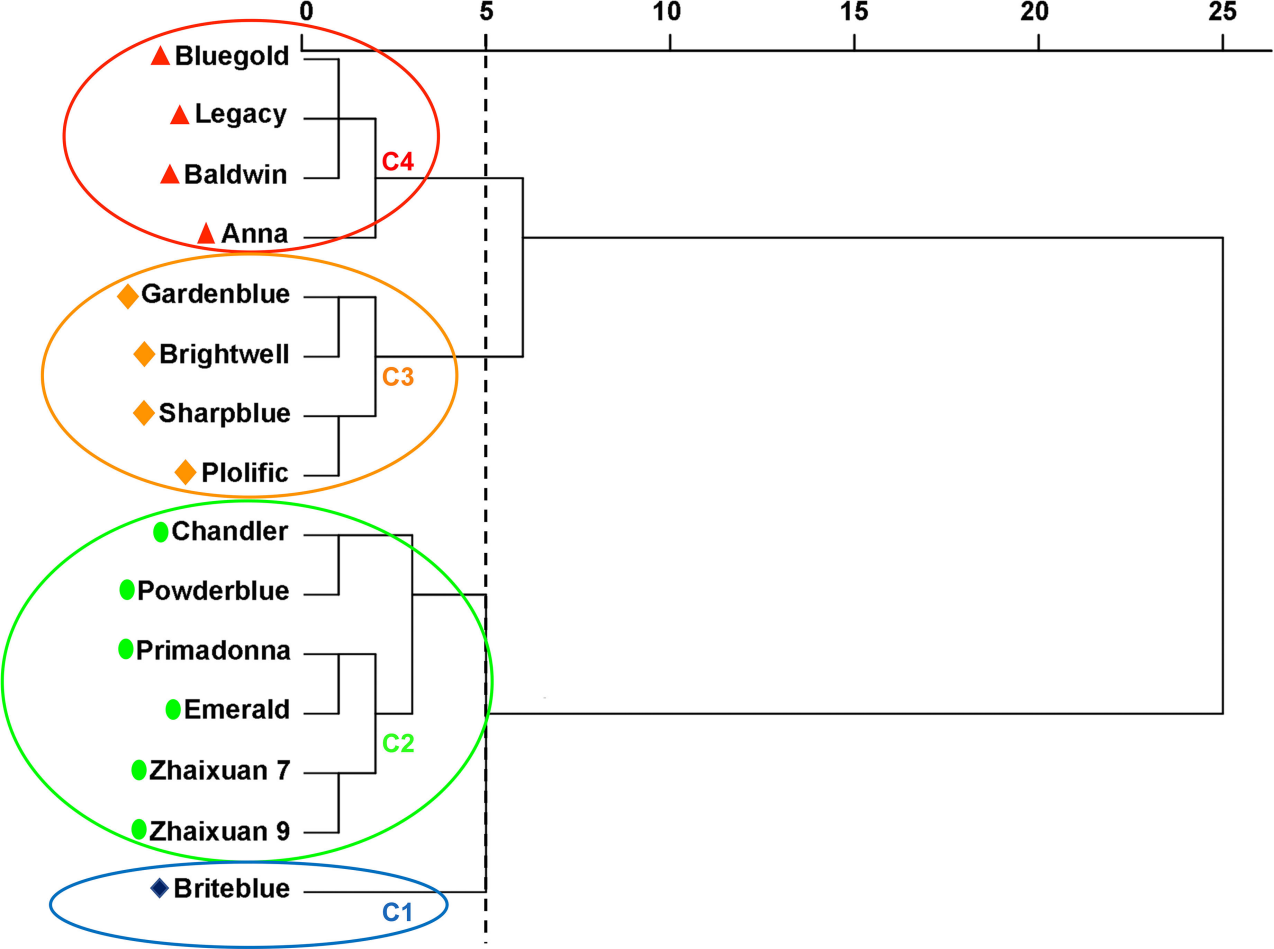
Euclidean distance cluster analysis to evaluate the resistance of 15 blueberry seedling cultivars to high soil pH stress. Blue circle C1 includes one high soil pH-tolerant blueberry cultivar, green circle C2 includes six intermediate high soil pH-tolerant blueberry cultivars, yellow circle C3 includes four low high soil pH-tolerant blueberry cultivars, and red circle C4 includes four high soil pH-sensitive blueberry cultivars.

**Figure 7:**
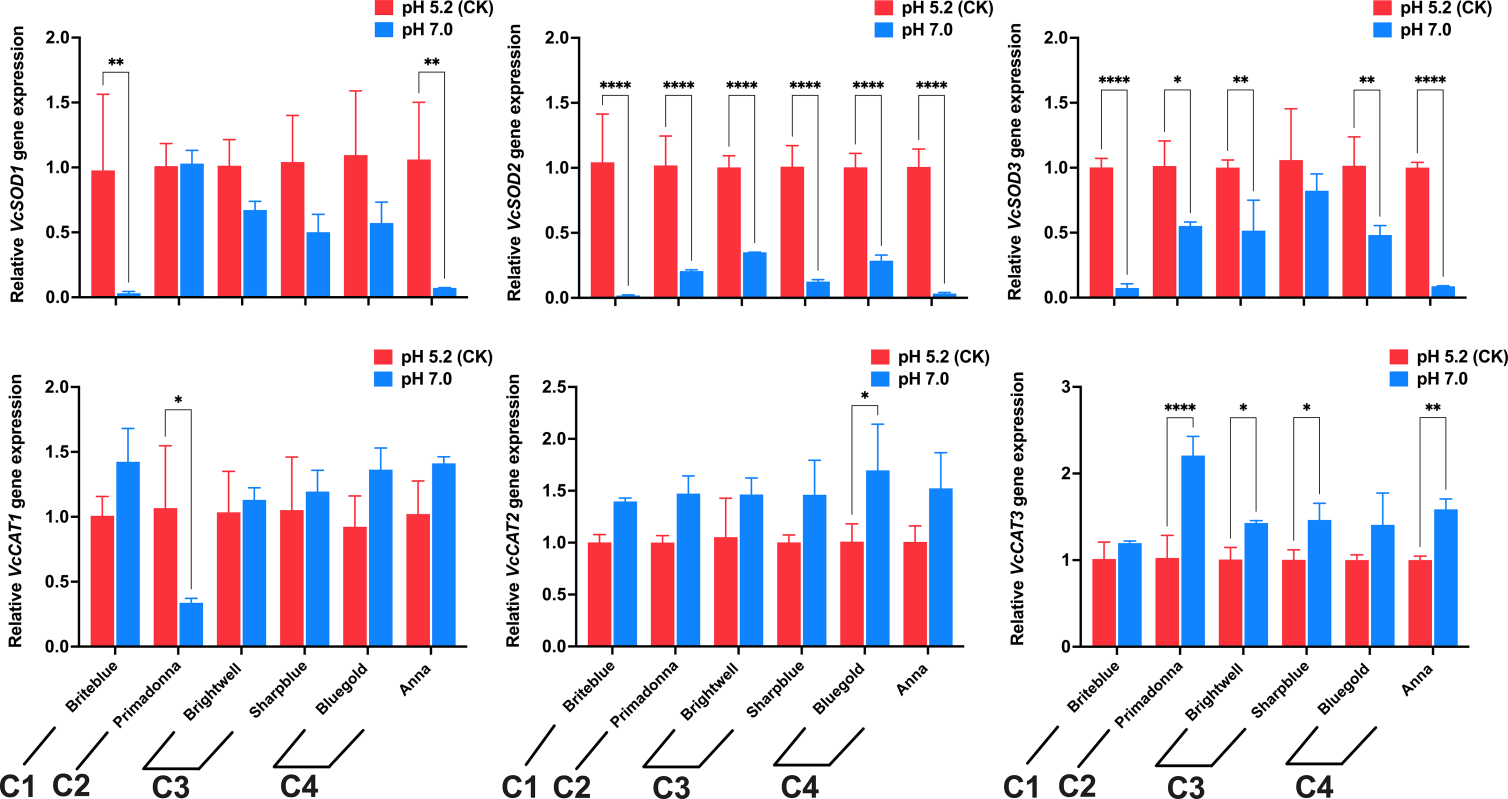
Effects of high soil pH stress on antioxidant system gene expression levels (*SOD* and *CAT*) in four types of high soil pH-tolerant blueberry leaves (6 cultivars). C1 represents high soil pH-tolerant blueberry cultivars, C3 represents low high soil pH-tolerant blueberry cultivars, C4 represents high soil pH-sensitive blueberry cultivars, and C2 represents intermediate blueberry cultivars. Values are expressed as the means ± SDs after 3 repetitions. * represents a significant difference at p<0.05, ** represents a significant difference at p<0.01, and **** represents a significant difference at p<0.0001, determined by Tukey’s test.

The Euclidean distance method and hierarchical clustering method were used to analyze the *D* value (**Figure 6**). The results showed that the 15 tested blueberry cultivars can be divided into four types based on a Euclidean distance of 5: the high soil pH tolerance type (‘Briteblue’), the intermediate type (‘Zhaixuan 9’, ‘Zhaixuan 7’, ‘Emerald’, ‘Primadonna’, ‘Powderblue’ and ‘Chandler’), the low high soil pH tolerance type (‘Brightwell’, ‘Gardenblue’, ‘Plolific’ and ‘Sharpblue’) and the high soil pH sensitivity type (‘Legacy’, ‘Bluegold’, ‘Baldwin’ and ‘Anna’). Overall, under high soil pH stress, the expression of the antioxidant-related enzyme genes *VcSOD1*, *VcSOD2* and *VcSOD3* in the four-tolerant blueberry types decreased, while the expression of *VcCAT1*, *VcCAT2* and *VcCAT3* increased (**Figure 7**), indicating that the antioxidant system had a certain effect on blueberry adaptation to stress.

Using the *D* value as the dependent variable and the relative ratio of the 16 indices as the independent variable, stepwise regression analysis was performed to establish the *D* regression equation of blueberry cultivar resistance to high soil pH stress: *D*=1.299+0.359X_1_+0.216X_2_+0.208X_3_+0.521X_4_+0.307X_5_+0.261X_6_+0.275X_7_ (R^2 =^ 0.997). X_1_, X_2_, X_3_, X_4_, X_5_, X_6_, and X_7_ represent plant height, SS, E, leaf length, C_i_, SOD, and SPAD, respectively. The above seven indices had significant effects on the *D* value of blueberry tolerance to high soil pH levels.

## Discussion

As one of the important physical and chemical properties of soil, soil pH is the key factor in controlling soil nutrient availability, soil microbial diversity and plant growth and development (Slessarev et al., 2016; Wielgusz et al., 2022; Yang et al., 2022). Different plants have different soil pH requirements. Blueberries are acid-loving soil plants, but high soil pH has been the primary abiotic stress factor limiting the growth of blueberry cultivation. Abiotic stress has adverse effects on plant phenotype, growth, biomass accumulation and yield (Holzapfel et al., 2004; Li et al., 2017; Sanoubar et al., 2020; Seleiman et al., 2021; Xu et al., 2022). Previous studies have shown that high soil pH levels cause iron deficiency and chlorosis in blueberry leaves, affect the absorption and transportation of mineral elements in plants, and inhibit plant growth and biomass accumulation (Jiang et al., 2017; Tamir et al., 2019; Tamir et al., 2021). High pH levels also affect the growth and development processes of blueberry flower bud differentiation and flowering phenology and reduce the yield and quality of fruits (Austin & Bondari, 1992; Yasuyuki et al., 2004; Kovaleski et al., 2015). Similar results were also found in our study. Compared with the CK treatment, the high soil pH stress treatment inhibited the growth of the 15 blueberry cultivars to different degrees (**Figure 2**), and leaf morphology showed different changes among cultivars (**Figure 3**). Overall, the growth inhibition of ‘Briteblue’ was low, while that of ‘Anna’ was the most severe. These results indicated differences in the tolerance of blueberry cultivars to high soil pH stress. Studies have confirmed that different blueberry cultivars have different tolerances to soil pH levels (Finn et al., 1993; Lyrene, 1997; Tsuda et al., 2014). Moreover, Paya-Milans et al. (2017) found that blueberry plants mainly adapt to a high soil pH environment by changing their nutrition, detoxification and cell wall gene networks and by effectively regulating related transcripts, as determined through transcriptome studies.

Abiotic stress not only affects the morphological growth of plants but also affects the photosynthesis and physiological metabolism of plants. Many studies have shown that plant physiological metabolism and photosynthesis are closely related to plant resistance (Chen et al., 2016; Arif et al., 2020; Nikoleta-Kleio et al., 2020; Li et al., 2022; Xiong et al., 2022). Osmotic regulation is an important physiological mechanism of plant stress resistance. Stress promotes the accumulation of cellular reactive oxygen species (ROS) and accelerates membrane lipid peroxidation to produce MDA and other toxic substances that destroy the cellular osmotic regulation system. MDA is an important indicator of the degree of damage plants experience under stress (Kaushik & Aryadeep, 2014; AbdElgawad et al., 2016). When osmotic balance is disrupted, plants maintain cellular osmotic pressure by accumulating substances, such as SS and SP (Xiong & Zhu, 2002; Kanu et al., 2019). In the present study, similar to other stress studies (Karimi & Salimi, 2021; Raza et al., 2022; Zhu et al., 2022a), the MDA content in the leaves of the 15 blueberry cultivars increased under the high soil pH stress conditions, and significant differences among the different cultivars (**Figure 4A**). The SS and SP contents in the leaves of most blueberry cultivars also increased to varying degrees (**Figures 4B, C**). These results indicate that different blueberry cultivars experienced different degrees of stress under high soil pH conditions and that blueberry cultivars can maintain the cell osmotic pressure balance through the accumulation of SSs and SPs, thereby ensuring the normal metabolic activity of their cells to strengthen their adaptation to stress. In addition, plants can eliminate excessive ROS by activating their own antioxidant mechanisms. SOD and CAT are the main components of the antioxidant enzyme system, and higher SOD and CAT activities are beneficial to the adaptation of plants to stress (Shams et al., 2016; Tian et al., 2020; Zhu et al., 2022b). Our study showed that CAT activity in the leaves of the different blueberry cultivars increased under high soil pH stress, whereas SOD activity was inhibited (**Figures 4D, E**). Meanwhile, under high soil pH stress, the *VcSOD1*, *VcSOD2* and *VcSOD3* gene expression levels in blueberry decreased, and the *VcCAT1*, *VcCAT2* and *VcCAT3* expression levels increased (**Figure 7**), which may be the main reason for the observed change in enzyme activity (Dionisio-Sese & Tobita, 1998). In addition, abiotic stress inhibits chlorophyll synthesis and reduces plant photosynthetic intensity, and damage to the plant photosynthetic system is related to osmotic adjustment substances and antioxidant enzyme systems (Efeoglu et al., 2009; Yousfi et al., 2010; Ying et al., 2015; Zhang et al., 2021). In the present study, the SPAD of blueberry leaves decreased significantly under the high soil pH stress treatment, and photosynthetic indices, such as P_n_, E, and G_s_, also significantly decreased. Significant differences were noted among the cultivars, indicating that the damage to the photosynthetic systems of blueberry cultivars differed under high soil pH stress. Photosynthesis in the blueberry cultivars ‘Climax’ and ‘Chaoyue NO. 1’ showed similar changes under high soil pH conditions (Jiang et al., 2019). In summary, the expression of the antioxidant genes *SOD* and *CAT* in blueberry leaves stimulated the accumulation of SS and SP and affected plant photosynthesis, which may potentially be a mechanism underlying the blueberry response to high soil pH stress.

The stress resistance capacity of plants is a product of the response to adverse environmental impacts and long-term evolution. Using a single index to evaluate the stress resistance of plants is not reliable, and it is more scientific and reasonable to use multidimensional indices to comprehensively evaluate plant stress resistance (Sloane et al., 1990). In addition, plants are a connected system, and a correlation exists between the measured resistance indicators. If an indicator is directly used for plant resistance analysis, then the accuracy of the results will be affected (Sun et al., 2021). Therefore, in our tudy, the HSTCs of the 16 growth and physiological indices related to blueberry resistance were used as an evaluation index to measure the ability of the blueberry cultivars to resist high soil pH levels. Correlation analysis was used to explain the relationship between each index, and PCA was used to transform multiple complex indices into a few independent and unrelated CIs. Based on this approach, the MF was used to comprehensively evaluate the soil pH tolerance of the different blueberry cultivars. The results obtained using the above analysis method are reliable and have been applied to the comprehensive evaluation of resistance in *Cucumis melo* L. (Weng et al., 2021), *Vitis vinifera* L. (Karimi & Salimi, 2021), *Prunussalicina* L. (Hamdani et al., 2021), *Populus deltoides* Marsh. (Chen et al., 2022), *Quercus* (Xiong et al., 2022), *Populus simonii × Populus nigra* (Liu et al., 2022) and other plants. Our study results revealed correlations among blueberry growth, osmotic adjustment substances, the antioxidant system, and the photosynthetic system under high soil pH conditions. PH and osmotic adjustment substances were significantly positively correlated with photosynthesis. SS and SP were significantly negatively correlated with blueberry leaf traits and MDA content; MDA was significantly negatively correlated with C_i_ but significantly positively correlated with P_n_ (**Figure 5**) (p<0.05) (Hura et al., 2007). Similar to the analysis results of (Sun et al., 2021), we obtained five PCs through PCA, accounting for 82.12% of the total variation in the 16 measurement indices. PC1 mainly reflects the morphological index and SP information, PC2 reflects the relevant photosynthesis information, PC3 reflects the correlations with MDA, and PC4 and PC5 mainly reflect the relevant SPAD and antioxidant enzyme information (**Table 3**). The above analyses showed that blueberries can adapt to a high soil pH environment through the synergistic effects of the osmotic adjustment system, antioxidant system, photosynthetic system and phenotypic changes. Subsequently, the weight of each CI was obtained according to the PCA, and the MF was used to comprehensively evaluate the tolerance of the blueberry cultivars to high pH soil. Resistance was ranked according to the *D* value (the greater the *D* value, the stronger the resistance), and the results are shown in **Table 4**. Then, according to the *D* value for the different blueberry cultivars, 15 blueberry cultivars were divided into four types: the high soil pH-tolerance type (‘Briteblue’), the intermediate tolerance type (‘Zhaixuan 9’, ‘Zhaixuan 7’, ‘Emerald’, ‘Primadonna’, ‘Powderblue’ and ‘Chandler’), the low high soil pH-tolerance type type (‘Brightwell’, ‘Gardenblue’, ‘Plolific’ and ‘Sharpblue’) and the soil pH-sensitive type (‘Legacy’, ‘Bluegold’, ‘Baldwin’ and ‘Anna’) (**Figure 6**).

The response of plants to stress is a complex physiological and metabolic process. In this study, stepwise regression analysis was performed using the *D* value and 16 measurement indices, and the corresponding linear equation was established (R^2 =^ 0.997). Seven indices (plant height, SS, E, leaf length, C_i_, SOD and SPAD) were selected and were found to be closely related to blueberry tolerance to high soil pH levels, and the analysis results were similar to those of (Finn et al., 1993), Tsuda et al. (2014); Xu et al. (2017) and Jiang et al. (2019). Thus, these indices can be used as reference identification indices for screening blueberry cultivars with high soil pH tolerance. In addition, the yield and quality of blueberry fruits are a direct embodiment of the economic value of blueberry plants (Ortega-Farias et al., 2021). A comparison of differences in fruit quality among the different resistant cultivars and the molecular mechanism of blueberry adaptability to high soil pH levels need to be further studied and explored.

## Conclusions

In this study, the 15 blueberry cultivars examined were divided into four categories after comprehensive evaluation. In these groups, ‘Briteblue’ was the most tolerant cultivar to high-pH soil, while ‘Anna’ was the most sensitive cultivar to high-pH soil. Through stepwise regression analysis, plant height, SS, E, leaf length, C_i_, SOD, and SPAD were selected to identify and predict the high-pH soil tolerance of blueberry cultivars. In the future, further research on the breeding of high soil pH tolerant blueberry cultivars and their tolerance mechanisms needs to be explored.

## Data availability statement

The original contributions presented in the study are included in the article/**Supplementary Material**. Further inquiries can be directed to the corresponding authors.

## Author contributions

HY and YW: Experimental design, interpretation of results and writing of the article. HY, YW and CZ: Performed the experiments. LL: Formal analysis. WW: Resources. WL: Writing – review & editing. All authors contributed to the article and approved the submitted version.
