# Supplementary material for: Comprehensive resistance evaluation of 15 blueberry cultivars under high soil pH stress based on growth phenotype and physiological traits
Source: Front Plant Sci. 2022 Dec 9;13:1072621. doi: 10.3389/fpls.2022.1072621 (PMC9780598; doi:10.3389/fpls.2022.1072621)
Supplement: Supplementary file 1 [file DataSheet_1.docx]

| Gene | Forward | Reverse |
| --- | --- | --- |
| *SOD1* | ATGCTGGCGGAAGGATTGCC | GCACGCACAACAACGAACGG |
| *SOD2* | GCACCTCCTCCGTTGAAGGC | CCCATGAGGCCCTGGAGTGA |
| *SOD3* | TGGCAGGGCCCTTGTTGTTC | CCACCTGCATTGCCAGTCGT |
| *CAT1* | TGCAGCGAGACAGAGCAAGC | TGGCGACCTTGGGAAGGAAGA |
| *CAT2* | GCTGCCTTTCGCTGAATCCGA | TCAACCGCATGACTGCTACGC |
| *CAT3* | GCCGATGGGTCGAAGCCTTG | GCCTCATGTTGAGCCGAGTGG |
| *NAPDH* | GGTTATCAATGATAGGTTTGGCA | CAGTCCTTGCTTGATGGACC |

Table S1 Primer sequences for the genes used in the quantitative real-time PCR analysis.

**Table S2** HSTCs of the growth and leaf physiological indices of 15 blueberry seedling cultivars under high soil pH stress.

| Cultivar | Type | PH | MD | CW | LL | LW | LT | SP | SS | MDA | CAT | SOD | SPAD | P_n_ | E | C_i_ | G_s_ |
| --- | --- | --- | --- | --- | --- | --- | --- | --- | --- | --- | --- | --- | --- | --- | --- | --- | --- |
| Baldwin | Rabbiteye blueberry  (RB) | 0.36  ±0.07e | 0.88  ±0.11ns | 0.50  ±0.12c | 0.76  ±0.02e-h | 0.61  ±0.04f | 0.72  ±0.01hi | 1.26  ±0.04de | 1.03  ±0.02ghi | 1.23  ±0.16fg | 1.23  ±0.16ef | 0.86  ±0.01cd | 0.61  ±0.07def | 0.43  ±0.06de | 0.15  ±0.03h | 1.07  ±0.04bc | 0.14  ±0.02i |
| Briteblue |  | 0.80  ±0.15ab | 0.93  ±0.10ns | 0.63  ±0.03abc | 0.90  ±0.06b-e | 0.93  ±0.07ab | 0.90  ±0.05cde | 1.07  ±0.13e | 1.41  ±0.03b | 1.25  ±0.04efg | 2.06  ±0.89cde | 0.83  ±0.01ef | 0.79  ±0.02ab | 0.57  ±0.01bc | 0.91  ±0.03b | 1.38  ±0.20a | 0.90  ±0.03c |
| Brightwell |  | 0.72  ±0.22abc | 0.79  ±0.19ns | 0.62  ±0.22abc | 0.92  ±0.11bcd | 0.89  ±0.10abc | 0.99  ±0.11bc | 1.19  ±0.05de | 1.02  ±0.08hi | 1.64  ±0.01c | 1.06  ±0.34f | 0.92  ±0.01a | 0.69  ±0.05b-f | 0.35  ±0.04e | 0.26  ±0.02g | 0.86  ±0.03f | 0.24  ±0.02h |
| Gardenblue |  | 0.42  ±0.09e | 0.75  ±0.17ns | 0.46  ±0.08c | 0.83  ±0.09c-g | 0.87  ±0.07a-d | 0.77  ±0.06fgh | 0.99  ±0.06e | 1.14  ±0.03ef | 1.98  ±0.09b | 2.25  ±0.79cd | 0.89  ±0.01b | 0.79  ±0.12ab | 0.47  ±0.06cd | 0.52  ±0.02de | 0.90  ±0.02def | 0.50  ±0.06ef |
| Powderblue |  | 0.66  ±0.14bcd | 0.81  ±0.14ns | 0.71  ±0.1ab | 0.96  ±0.01abc | 0.98  ±0.03a | 0.79  ±0.02fgh | 1.25  ±0.13de | 1.20  ±0.04e | 1.87  ±0.01b | 6.35  ±0.92a | 0.83  ±0.01ef | 0.75  ±0.05abc | 0.57  ±0.03bc | 0.48  ±0.05e | 0.93  ±0.10def | 0.46  ±0.04f |
| Plolific |  | 0.48  ±0.05de | 0.70  ±0.06ns | 0.50  ±0.13c | 0.69  ±0.02gh | 0.70  ±0.07ef | 0.91  ±0.11cde | 1.00  ±0.01e | 1.39  ±0.03bc | 1.42  ±0.19de | 3.22  ±0.33b | 0.85  ±0.01cde | 0.83  ±0.05a | 0.44  ±0.04de | 0.34  ±0.06f | 0.88  ±0.07ef | 0.28  ±0.01gh |
| Anna | Southern highbush blueberry  (SHB) | 0.38  ±0.09e | 0.73  ±0.11ns | 0.50  ±0.04c | 0.67  ±0.02h | 0.69  ±0.05ef | 0.73  ±0.05hi | 4.29  ±0.28b | 1.00  ±0.02i | 1.65  ±0.09c | 1.65  ±0.09c-f | 0.72  ±0.01h | 0.60  ±0.07ef | 0.57  ±0.06bc | 0.54  ±0.05d | 0.92  ±0.08def | 0.51  ±0.03ef |
| Primadonna |  | 0.53  ±0.05de | 0.77  ±0.09ns | 0.46  ±0.14c | 0.67  ±0.07h | 0.73  ±0.04ef | 0.81  ±0.04e-h | 10.05  ±2.08a | 1.33  ±0.04cd | 1.28  ±0.11efg | 1.28  ±0.11ef | 0.87  ±0.04bcd | 0.74  ±0.06abc | 0.60  ±0.16b | 1.13  ±0.01a | 1.19  ±0.11b | 1.12  ±0.01a |
| Sharpblue |  | 0.44  ±0.07e | 0.82  ±0.05ns | 0.58  ±0.23abc | 0.86  ±0.03c-f | 0.80  ±0.01b-e | 0.86  ±0.16d-g | 1.30  ±0.19de | 1.11  ±0.07fg | 2.31  ±0.29a | 1.50  ±0.50def | 0.81  ±0.02f | 0.77  ±0.04ab | 0.62  ±0.09b | 0.49  ±0.01e | 0.73  ±0.12g | 0.46  ±0.02f |
| Zhaixuan 7 |  | 0.79  ±0.12ab | 0.79  ±0.09ns | 0.55  ±0.07abc | 0.78  ±0.08d-h | 0.79  ±0.05cde | 1.03  ±0.05b | 1.07  ±0.10e | 1.29  ±0.03d | 1.39  ±0.07def | 1.34  ±0.29ef | 0.88  ±0.01bc | 0.71  ±0.02a-e | 0.57  ±0.01bc | 0.92  ±0.04b | 1.17  ±0.08b | 1.00  ±0.06b |
| Zhaixuan 9 |  | 0.89  ±0.25a | 0.99  ±0.17ns | 0.74  ±0.08a | 1.09  ±0.18a | 0.94  ±0.16a | 0.89  ±0.05c-f | 1.01  ±0.05e | 1.01  ±0.02hi | 1.14  ±0.05g | 1.21  ±0.11ef | 0.83  ±0.02ef | 0.73  ±0.06abc | 0.39  ±0.01de | 0.53  ±0.02de | 1.08  ±0.07bc | 0.54  ±0.07e |
| Bluegold | Northern highbush blueberry  (NHB) | 0.53  ±0.15cde | 0.90  ±0.09ns | 0.55  ±0.14abc | 0.73  ±0.08fgh | 0.73  ±0.05def | 0.93  ±0.06bcd | 2.30  ±0.42c | 1.09  ±0.02fgh | 1.23  ±0.03fg | 1.23  ±0.03ef | 0.71  ±0.02h | 0.57  ±0.05f | 0.44  ±0.07de | 0.33  ±0.02f | 0.93  ±0.02def | 0.31  ±0.01g |
| Chandler |  | 0.45  ±0.2e | 0.81  ±0.30ns | 0.54  ±0.10bc | 0.90  ±0.19b-e | 0.98  ±0.15a | 0.65  ±0.03i | 2.02  ±0.50cd | 1.68  ±0.04a | 1.14  ±0.07g | 2.42  ±1.32bc | 0.77  ±0.02g | 0.78  ±0.08ab | 0.35  ±0.03e | 0.37  ±0.04f | 0.99  ±0.01cde | 0.31  ±0.04g |
| Emerald |  | 0.55  ±0.07cde | 0.79  ±0.05ns | 0.74  ±0.05ab | 1.04  ±0.09ab | 0.96  ±0.12a | 0.77  ±0.04gh | 1.04  ±0.19e | 1.28  ±0.14d | 1.48  ±0.12cd | 1.91  ±0.16c-f | 0.78  ±0.01g | 0.72  ±0.08a-d | 0.80  ±0.05a | 0.84  ±0.07c | 1.02  ±0.03cd | 0.82  ±0.09d |
| Legacy |  | 0.37  ±0.11e | 0.81  ±0.11ns | 0.47  ±0.07c | 0.81  ±0.09d-h | 0.90  ±0.05abc | 1.31  ±0.11a | 0.85  ±0.05e | 1.11  ±0.03f | 1.84  ±0.14b | 2.29  ±0.30cd | 0.85  ±0.02de | 0.63  ±0.12def | 0.61  ±0.05b | 0.34  ±0.01f | 0.83  ±0.03fg | 0.31  ±0.02g |

Note: PH, plant height; MD, main stem diameter; CW, crown width; LL, leaf length; LW, leaf width; LT, leaf thickness; SP, soluble protein; SS, soluble sugar; MDA, malondialdehyde; SOD, superoxide dismutase; CAT, catalase; SPAD, chlorophyll relative content; P_n_, net photosynthetic rate; E, transpiration rate; C_i_, intercellular CO_2_ concentration; G_s_, stomatal conductance. Different letters in the same column/trait indicate significant differences between different cultivars (p<0.05).
